# Diagnostic biomarker panels of osteoarthritis: UPLC-QToF/MS-based serum metabolic profiling

**DOI:** 10.7717/peerj.14563

**Published:** 2023-01-13

**Authors:** Xinxin Lin, Shiqi He, Suyu Wu, Tianwen Zhang, Sisi Gong, Tang Minjie, Yao Gao

**Affiliations:** 1 The School of Medical Technology and Engineering, Fujian Medical University, Fuzhou, China; 2 Fujian Fishery Resources Monitoring Center, Fuzhou, China; 3 Department of Laboratory Medicine, the Second Affiliated Hospital of Fujian Medical University, Quanzhou, China; 4 Department of Laboratory Medicine, the First Affiliated Hospital of Fujian Medical University, Fuzhou, China

**Keywords:** Osteoarthritis, Biomarker, UPLC-QToF/MS, Early diagnosis, Metabolic pathway analysis

## Abstract

Osteoarthritis (OA) is the most common joint disease in the world, characterized by pain and loss of joint function, which has led to a serious reduction in the quality of patients’ lives. In this work, ultrahigh performance liquid chromatography coupled with quadrupole time-of-flight tandem mass spectrometry (UPLC-QToF/MS) in conjunction with multivariate pattern recognition methods and an univariate statistical analysis scheme were applied to explore the serum metabolic signatures within OA group (*n* = 31), HC (healthy controls) group (*n* = 57) and non-OA group (*n* = 19) for early diagnosis and differential diagnosis of OA. Based on logistic regression analysis and receiver operating characteristic (ROC) curve analysis, seven metabolites, including phosphatidylcholine (18:0/22:6), p-cresol sulfate and so on, were identified as critical metabolites for the diagnosis of OA and HC and yielded an area under the curve (AUC) of 0.978. The other panel of unknown m/z 239.091, phosphatidylcholine (18:0/18:0) and phenylalanine were found to distinguish OA from non-OA and achieved an AUC of 0.888. These potential biomarkers are mainly involved in lipid metabolism, glucose metabolism and amino acid metabolism. It is expected to reveal new insight into OA pathogenesis from changed metabolic pathways.

## Introduction

Osteoarthritis (OA), whose pathological hallmarks are the loss of articular cartilage, the hypertrophic differentiation of chondrocytes, subchondral bone thickening, synovial inflammation and osteophyte formation (Li et al., 2018), which manifest as joint stiffness, pain and dysfunction, is the most prevalent form of arthritis and a major source of joint pain and disability (Mathiessen & Conaghan, 2017). There are multiple factors thought to be associated with OA, including aging, previous joint injury, obesity, genetics, sex and anatomical factors, however, the exact etiological mechanism has not yet been fully elucidated (Loeser, Collins & Diekman, 2016; Prieto-Alhambra et al., 2014). Non-steroidal anti-inflammatory drugs and intra-articular steroid injections are usually used to relieve the pain and inflammation of osteoarthritis clinically, but their efficacy is limited and toxicity is also great (Mathiessen & Conaghan, 2017). OA has affected over 250 million individuals worldwide (Carlson et al., 2019) and emerging evidence suggested that this number would steadily rise, which not only impacts people’s quality of life but also imposes a substantial socioeconomic burden (Mathiessen & Conaghan, 2017; Zhai, Randell & Rahman, 2018).

At present, early diagnosis is paramount but difficult for OA. The radiography and clinical manifestations are the main diagnostic methods. However, OA is diagnosed at the onset of clinical symptoms typically occurs when structural damage has been ineluctable (Carlson et al., 2018). More seriously, distinguishing OA from other arthritic diseases has become challenging because of the similar clinical symptoms and similar pathological features (Jiang et al., 2013). Common manifestations of arthritic diseases are inflammation, functional degradation of connective tissues, along with pain and stiffness (Shim et al., 2018). These difficulties not only bring about a great confusion in diagnosis but also delay the use of optimal therapy. Taken together, there are still a lack of reliable markers with high sensitivity and specificity for the early and accurate diagnosis of OA, which highlight the desire to discover specific biomarkers.

Metabolomics is an emerging field which reflects the metabolic response of living systems to pathophysiological stimuli or environmental conditions (Zeng et al., 2017), and focuses on the low-molecular-weight metabolites to detect potential diagnostic biomarkers. Currently, nuclear magnetic resonance spectroscopy and mass spectrometry (MS) coupled with gas chromatography or liquid chromatography (LC) are the most common analytical tools in detection of metabolites and have been widely used in various clinical metabolomics studies (Hackshaw et al., 2019; Yang et al., 2018a). Among these techniques, LC shows satisfactory complex matrix separation and MS displays high sensitivity, resolution and reproducibility. And LC coupled with MS allows to attain the most comprehensive coverage of metabolic features (Han et al., 2019), which is more suitable for metabolite screening. So far, this well-established platform has been widely applied in many disease researches such as ovarian cancer, acute myeloid leukemia and breast cancer (Shao et al., 2016; Wang et al., 2019; Yang et al., 2018b). In addition, serum could be an ideal biomedium for metabolic profile study since it is relatively common, more accessible, more stable and less invasive than other biosamples. Compared with whole blood and plasma, serum components are unaffected by cellular components and hemolytic factors. In contrast to urine, serum is insusceptible to dietary factors and sampling time. Moreover, the storage conditions of serum are relatively simple, which can not only be stored at room temperature for a short time, but also have no great impact on most tests when stored at −80 °C for a long time. More importantly, serum can provide a snapshot of metabolic dynamics and intuitively reflect the alters in endogenous marker concentrations, helping to excavate deeper into the pathogenesis of the diseases (Yang et al., 2017). Therefore, serum metabolomics is still the mainstream tool for biomarker discovery in many clinical researches (Dong et al., 2015; Khan et al., 2019; Pan et al., 2019).

Considering that systemic metabolic dys-regulation has taken place in the pathogenesis of OA (June et al., 2016), if the molecular metabolites could be identified before irreversible degeneration happened, it would be helpful to make decisions in clinical diagnosis to delay OA progression and minimize the negative effects in society. Hence, ultrahigh performance liquid chromatography coupled with quadrupole time-of-flight mass spectrometry (UPLC-QToF/MS) combined with univariate and multivariate statistical analysis was carried out to identify a distinct serum metabolic signature to robustly discriminate OA patients from healthy controls (HC), as well as non-osteoarthritis (non-OA) cohort. The primary objective of this study was to explore the differentially expressed serum metabolites of OA which could be helpful for diagnosis at an early stage. Besides that, we aimed to better understand the dysfunction mechanism of OA at the metabolic level.

## Materials and Methods

### Study design

Ethics approval was obtained from the Ethics Committee of Fujian Medical University (No. 2019[34]). In this study, a total of 107 participants were recruited in 2019 at the First Affiliated Hospital of Fujian Medical University. Written informed consent signed by each of participants was obtained prior to blood samples were taken. Based on the international consensus diagnostic criteria, 31 patients with OA as OA group and 19 patients with different forms of arthritis as non-OA group, including 11 rheumatoid arthritis, three ankylosing spondylitis, two gout, one psoriatic arthritis, one septic arthritis and one psoriasis, were enrolled. And healthy control (HC) group was constituted by 57 healthy volunteers with no declared history of arthritis. The general characteristics of the subjects in each group were summarized in Table 1 and all groups were matched by age and gender.

**Table 1:** Demographic characteristics of recruited subjects.

	OA	Non-OA	HC
Number of subjects	31	19	57
Age (mean ± SD)	53.38 ± 14.72	50.05 ± 14.59	49.81 ± 12.94
Gender (male/female)	10/21	10/9	26/31

**Note:**

SD, standard deviation; OA, osteoarthritis; non-OA, non-osteoarthritis; HC, healthy control.

All serum samples were collected by venipuncture and were immediately separated by centrifuging at a speed of 3,000 g for 10 min at 4 °C. Fractions (200 μL) of the serum supernatants were quickly stored at −80 °C until the UPLC-QToF/MS analysis was conducted.

### Chemicals and reagents

High performance liquid chromatography (HPLC) grade methanol and acetonitrile were obtained from Merck (Darmstadt, Germany). Analytical grade formic acid and HPLC grade ammonium acetate were purchased from ROE Scientific inc (Newark, DE, USA). Leucine enkephalin (LE) was purchased from Sigma-Aldrich (St. Louis, MO, USA). *L*-2-chlorophenylalanine and standards for metabolite identification were bought from Shanghai Aladdin Bio-Chem Technology Co., LTD. A Milli-Q purification system (Bedford, MA, USA) was used to provide ultra-pure water. A total of 2.0-mL vials were purchased from Agilent (Palo Alto, CA, USA).

### Serum sample preparation

Before analysis, each serum sample was thawed at 4 °C and immediately centrifuged at 20,000 g for 10 min. Subsequently, each 100 μL aliquot of serum was added with 400 μL extraction solvents, namely methanol-acetonitrile (50:50, v/v) containing L-2-chlorophenylalanine (0.6 μg/ml) as internal standard (IS) in a 2.0-ml Eppendorf (EP) tube. The mixture was vigorously shaken for 30 s, refrigerated at −20 °C for 20 min and then centrifuged at 20,000 g for 15 min. Afterwards, an aliquot of 200 μL of supernatant was transferred to a 1.5-ml EP tube prior to centrifuging at 30,000 g for 5 min. Finally, 150 μL of supernatant was transferred into the 2.0-mL vial for UPLC-QToF/MS analysis.

To ensure consistent condition and reliability of this analytical system, quality control (QC) sample was obtained by mixing equal aliquots (10 μL) of each individual sample and pretreated as serum sample preparation (Acera et al., 2019). The pooled QC sample was injected six times at the start of the analytical batch to balance the column, and once after every 10 injections of serum samples throughout the analytical workflow. Therefore, QC sample was analyzed for a total of 17 times during the whole analysis process.

### UPLC-QToF/MS conditions

In the present study, a Waters ACQUITY ultrahigh performance liquid chromatography system (Waters, Milford, MA, USA) coupled with Xevo G2-S QToF tandem mass spectrometer (Waters, Milford, MA, USA) with electrospray ionization (ESI) was used to perform the analysis. All the pretreatment serum samples were kept at 4 °C during the experimental process and 2 μL was injected into the Ethylene Bridged Hybrid (BEH)-C18 column (2.1 × 50 mm × 1.7 μm, Waters, Milford, MA, USA). In ESI positive ion mode (ESI+), mobile phase A was 0.1% aqueous formic acid and mobile phase B was 0.1% formic acid methanol, while in ESI negative ion mode (ESI−), mobile phase A was 5 mmol/L aqueous ammonium acetate and mobile phase B was 5 mmol/L ammonium acetate methanol. The column oven and the flow rate was set at 30 °C and 0.4 mL/min, respectively. The gradient elution program of this analysis method was set as follows: 0–0.5 min, B: 5%; 0.5–0.7 min, B: 5–80%; 0.7–5 min, B: 80–98%; 5–13.5 min, B: 98%; 13.5–14 min, B: 98–5%; 14–17 min, B: 5%.

The mass spectrometer was operated in both ESI+ and ESI−, and the mass range was set at 50–1,000 mass-to-charge ratio (m/z) to acquire the data. The optimal capillary voltage was set at 3.0 kV (ESI+) or 2.5 kV (ESI−), with sample cone voltage at 35 V (ESI+) or 40 V (ESI−). The source temperature was set at 120 °C (ESI+) or 100 °C (ESI−) and desolvation temperature was set at 450 °C. The gas flow for cone and desolvation were set to 50 and 650 L/h, respectively. In an attempt to calibrate mass accurately and monitor the signal in real time, LE was used as the reference compound (m/z 556.2771 in ESI+ and 554.2615 in ESI−) at a concentration of 1 μg/ml under a flow rate of 10 μl/min, and the lock spray frequency was set at 10 s for real-time accurate mass correction. The data were collected in centroid data mode with a scan time of 0.5 s. The MS^E^ mode was applied for the acquisition of the MS/MS spectra of representative fragments, where two acquisition functions with different collision energies were established including low collision energy of 20 V and the high collision energy of 30 V.

### Data analysis

The raw data from UPLC-QToF/MS were initially converted in MarkerLynx Applications Manager version 4.1 (Waters, Milford, MA, USA) for peak finding, filtering, and alignment. The main set-up parameters (Sun et al., 2016) were as follows: retention time (RT) range 0–14 min; mass range 50–1,000; extracted ion chromatograms (XIC) window 0.02 Da; RT window 0.2 min; mass window 0.02 Da; marker intensity threshold 2,500 counts; noise elimination level 6. And then the three-dimensional matrix data were generated, consisting of the sample name, the RT and m/z pair, and the ions peak areas corrected with LE. The observed m/z of every compound was corrected online. The data were subsequently exported to Microsoft Office Excel 2007 for IS peak area normalization and for removing the missing values by the 80% rule (Dong et al., 2015).

Thereafter, the pretreated data were transferred to Simca-P software (version 14.1; Umetrics AB, Malmö, Sweden) (Tu et al., 2021) which is mainly used for the statistical methods of principle component analysis (PCA) and partial least square (PLS) regression. PCA (Trygg, Holmes & Lundstedt, 2007), an unsupervised method, was initially applied to acquire an unbiased overview of the entire samples. Supervised model was subsequently carried out by orthogonal partial least squares-discriminant analysis (OPLS-DA), which is mainly focused on maximizing the distance between groups and has the capability to identify changed endogenous molecules (Gao et al., 2012). Pareto scaling was performed to center and scale the variance (Yang et al., 2018a). The quality of the fitting model was evaluated by parameter *R*^*2*^*Y* that was used to explain the percentages of y-variables, and parameter *Q*^*2*^ that represents the capacity of predictive value (Ma & Niu, 2021). Moreover, in order to guard against model over-fitting and acquire higher data fidelity for biomarker screening, permutation tests were performed 100 times. The metabolites that contributed to the classification were obtained according to variable importance in the projection (VIP) values from the OPLS-DA model which describes the overall contribution of each variable to the model, and those metabolites with higher VIP values represented more significant influence on differentiation among groups (Liu et al., 2019). In this study, the criterion of VIP value was artificially set to four to narrow down the candidates’ range and improve the accuracy of screening for potential biomarkers.

To further evaluate the differences of preselected metabolites between HC/OA/non-OA groups, the nonparametric univariate statistical analysis approach without requirement for sample normal distribution, Kruskal-Wallis test was executed by Statistical Product and Service Solutions (SPSS) 17.0 (IBM Corp., Armonk, NY, USA) (Gündüzöz et al., 2017). This statistical significance evaluation was conducted by comparison their normalized peak area between OA and HC/non-OA groups. Metabolite features with a *p*-value less than 0.05 were considered statistically significant in these analyses. Therefore, the range of the potential biomarkers of interest from preselected metabolites has been further narrowed.

To further ascertain whether those potential biomarkers have the diagnostic ability, receiver operating characteristic (ROC) curve was plotted and the area under the curve (AUC) was also computed *via* numerical integration of the curve by SPSS 17.0 software. Markers with AUC value greater than 0.8 had superior diagnostic performance (Alizadeh-Sedigh et al., 2022; Choe et al., 2022; Decraecker et al., 2022; Saleem et al., 2017). In addition, logistic regression analysis was simultaneously carried out to integrate these potential biomarkers. And the optimal AUC, sensitivity and specificity were determined using the maximum of the Youden index, which calculated as follows: Youden’s index = sensitivity + specificity − 1 (Alfitian et al., 2022; Hatakenaka et al., 2022; Marty et al., 2013).

### Identification of potential biomarkers and metabolic pathway analysis

For putative identification, the metabolites’ structural information were compared to the online databases (Fernández-García et al., 2018), including Human Metabolome Database (HMDB, http://www.hmdb.ca/) (Wishart et al., 2013) and METLIN (https://metlin.scripps.edu/index.php) (Smith et al., 2005) within 10 ppm mass accuracy. The robustness of molecular identification was further confirmed by matching with authentic standards based on tandem mass spectra.

For estimation of disturbed pathways subject to OA, the metabolic pathway analysis was executed by Kyoto Encyclopedia of Genes and Genomes (KEGG, http://www.genome.jp/kegg/) (Lin et al., 2021) based on the potential biomarkers from this study.

## Results

### Data quality assessment

**Figure 1:**
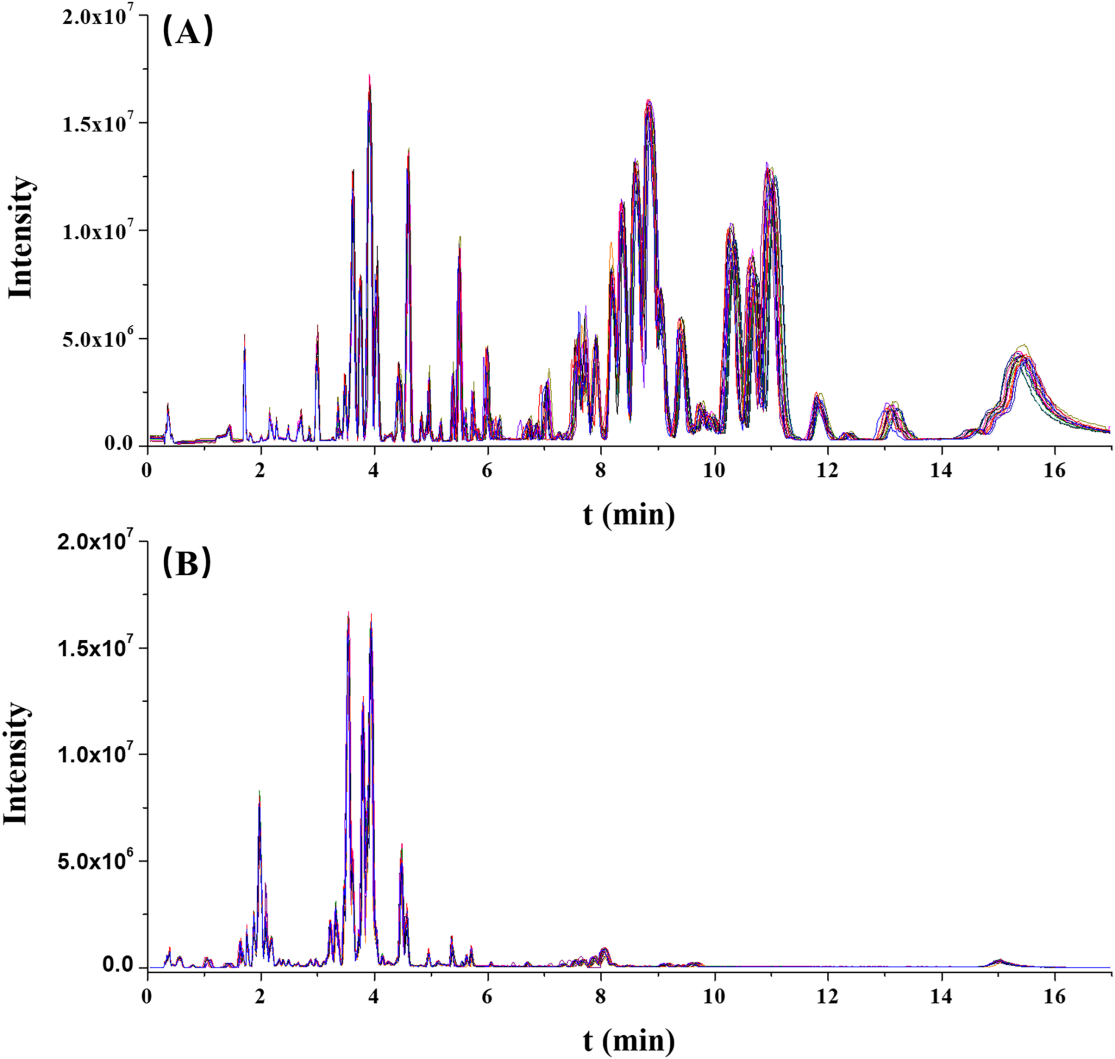
The overlaps of base peak intensity (BPI) chromatograms from the 17 quality control (QC) injections in (A) positive electrospray ionization (ESI) and (B) negative ESI modes, respectively.

The stability of this analytical run was preliminarily assessed by overlapping the chromatographic peaks from the QC results. As illustrated in Fig. 1, the base peak intensity (BPI) chromatograms were well overlapped, indicating a good reliability of this method.

Furthermore, the RT and normalized peak areas of five XIC peaks in two ionization modes from 17 injections of the QC sample were concerned. The fluctuation ranges of RT and normalized peak areas of these five ions were then calculated and shown by relative standard deviations (RSDs) in Table 2. The RSDs ranges of RT and normalized peak areas were 0.00–1.16% and 4.92–9.95%, respectively. In two ionization modes, the reproducibilities of RT and normalized peak areas were acceptable and collectively highlighted the robustness of this metabolomic platform.

**Table 2:** Reproducibility of the analytical method from five ions of the QC sample in positive and negative ionization modes (*n* = 17).

ESI mode	m/z	RT, min		Normalized peak area	
		Mean	RSD (%)	Mean	RSD (%)
ESI+	188.071	1.72	0.00	9.82	4.92
ESI+	365.245	4.83	0.19	3.26	9.80
ESI+	541.122	5.99	0.42	12.30	9.83
ESI+	734.571	10.01	1.15	5.30	9.25
ESI+	878.587	13.08	1.16	8.34	9.95
ESI−	187.005	1.79	0.98	11.42	7.77
ESI−	564.331	3.61	0.48	46.55	8.43
ESI−	568.362	4.57	0.19	31.62	8.99
ESI−	745.546	6.70	0.20	4.79	9.65
ESI−	802.560	8.05	0.28	26.24	9.90

**Note:**

ESI+, positive ESI mode; ESI−, negative ESI mode; RT, retention time; RSD, relative standard deviation.

### Overall serum metabolomic analysis

**Figure 2:**
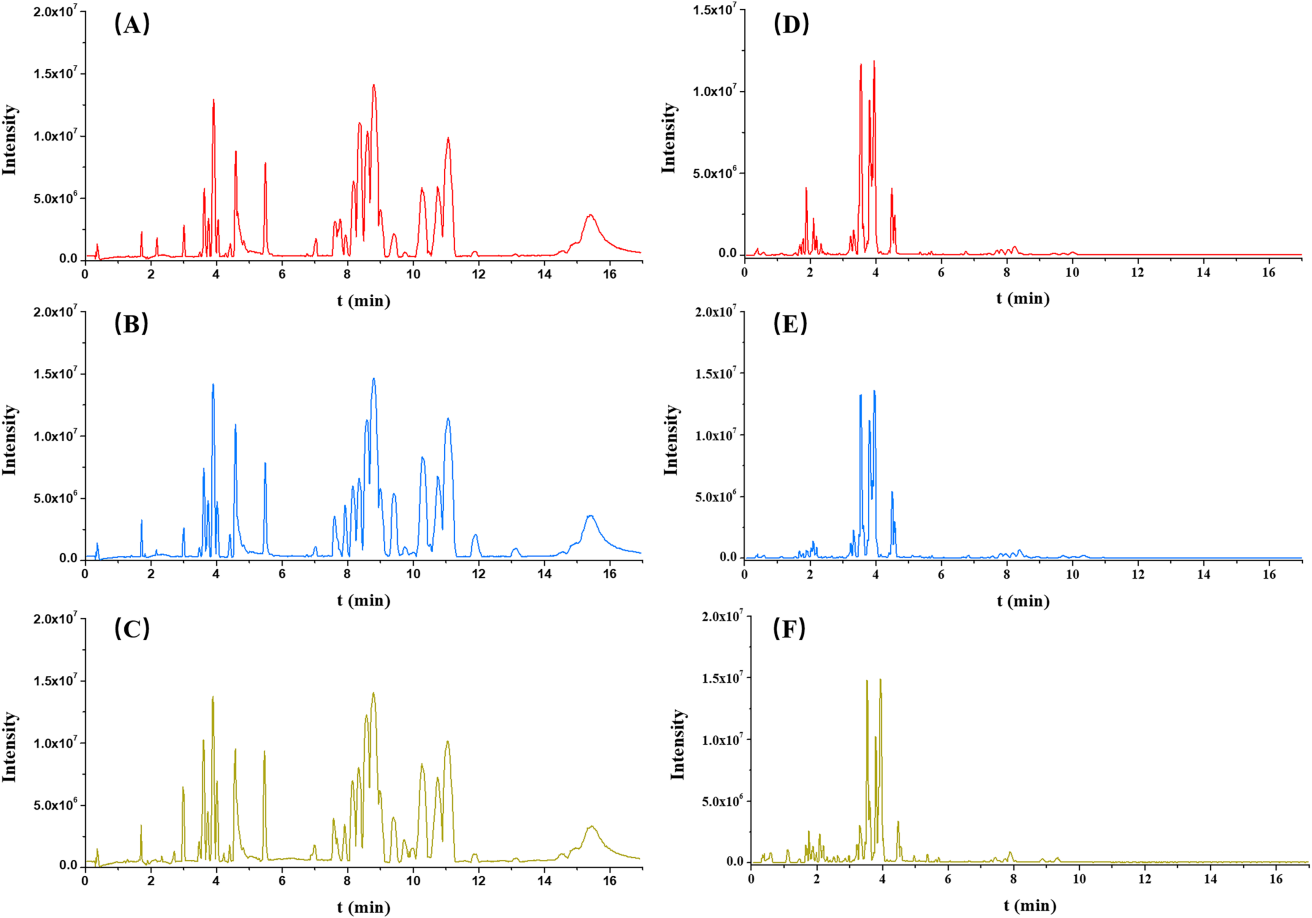
Typical serum intensity (BPI) chromatograms in OA (red), HC (green) and non-OA (yellow) groups. (A) OA, (B) HC and (C) non-OA groups in ESI+. (D) OA, (E) HC and (F) non-OA groups in ESI−.

Each serum sample was analyzed by untargeted metabolomics-based UPLC-QToF/MS, and the typical BPI chromatograms from three groups (OA, non-OA and HC) from both ESI+ and ESI− were presented in Fig. 2.

**Figure 3:**
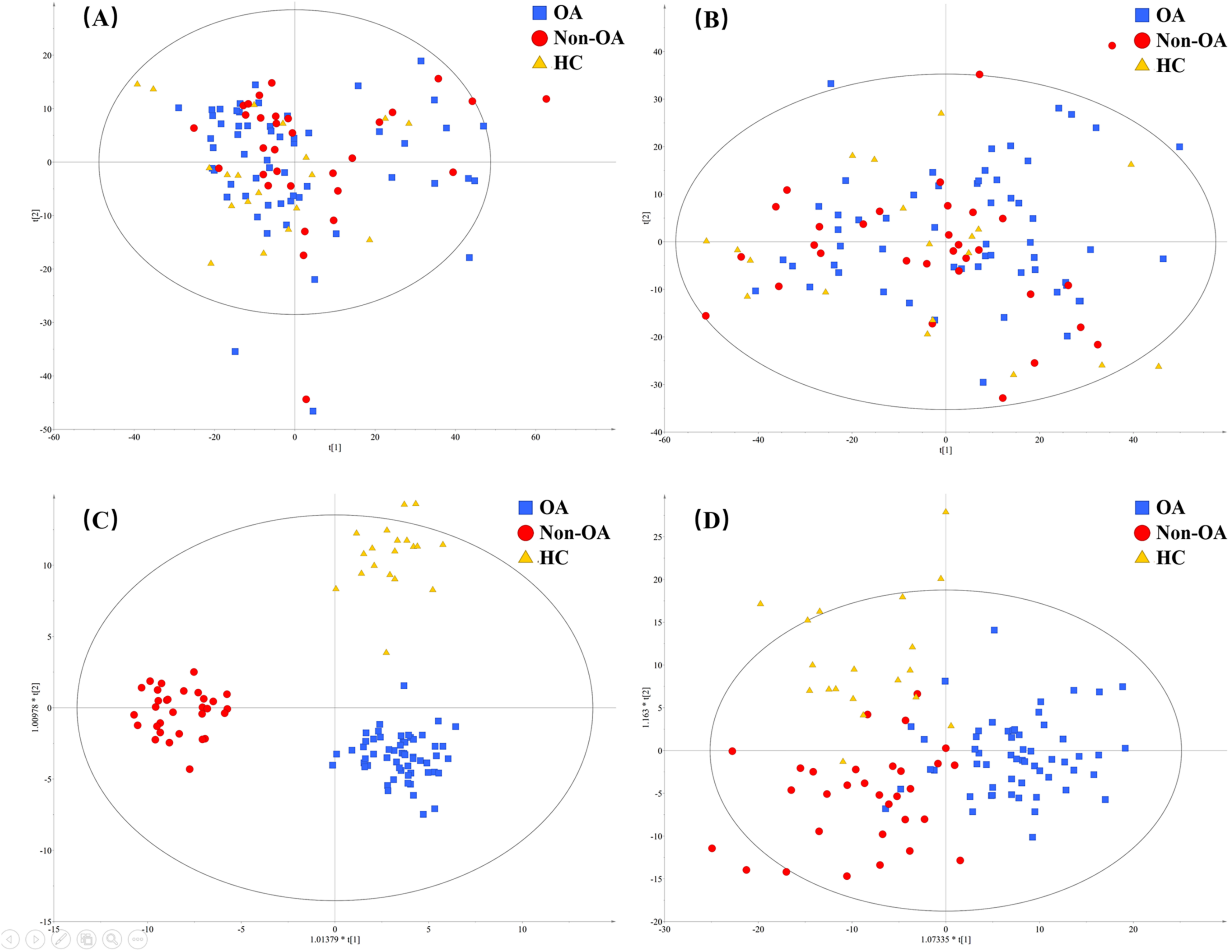
The PCA and OPLS-DA 3D score plots incorporating all the three groups. PCA models from (A) positive and (B) negative ionization modes; OPLS-DA models from (C) positive and (D) negative ionization modes. Red, green and yellow color represent OA, HC and non-OA samples, respectively. PCA, principal component analysis; OPLS-DA, orthogonal partial least squares-discriminant analysis; 3D, three dimensional.

A total of 4,577 (ESI+) and 5,804 (ESI−) metabolic features in the current data were obtained from UPLC-QToF/MS analysis. The multivariate statistical analyses were introduced to build metabolic profiling of OA, HC and non-OA groups. Preliminarily, based on the metabolite spectra of three groups samples, namely OA *vs* HC *vs* non-OA, the PCA 3-dimensional (3D) score plots in ESI+ and ESI− were obtained and depicted in Figs. 3A and 3B, respectively. However, distinct discrimination within groups could not be observed.

In an attempt to get better distinct discrimination, supervised OPLS-DA models were then applied. The OPLS-DA 3D score plots of two ionization modes were illustrated in Figs. 3C and 3D, presenting a more explicit group classification. The explained variation parameter *R*^*2*^*Y* and the cross-validation parameter *Q*^*2*^ of OPLS-DA models were 0.918 and 0.580 in ESI+, and 0.586 and 0.372 in ESI−, displaying a satisfactory separating tendency. These results implied that OA patients had the characteristics of serum metabolism and it is feasible to seek underlying biomarkers.

### OA-related metabolites screening

#### Metabolites for OA diagnosis

**Figure 4:**
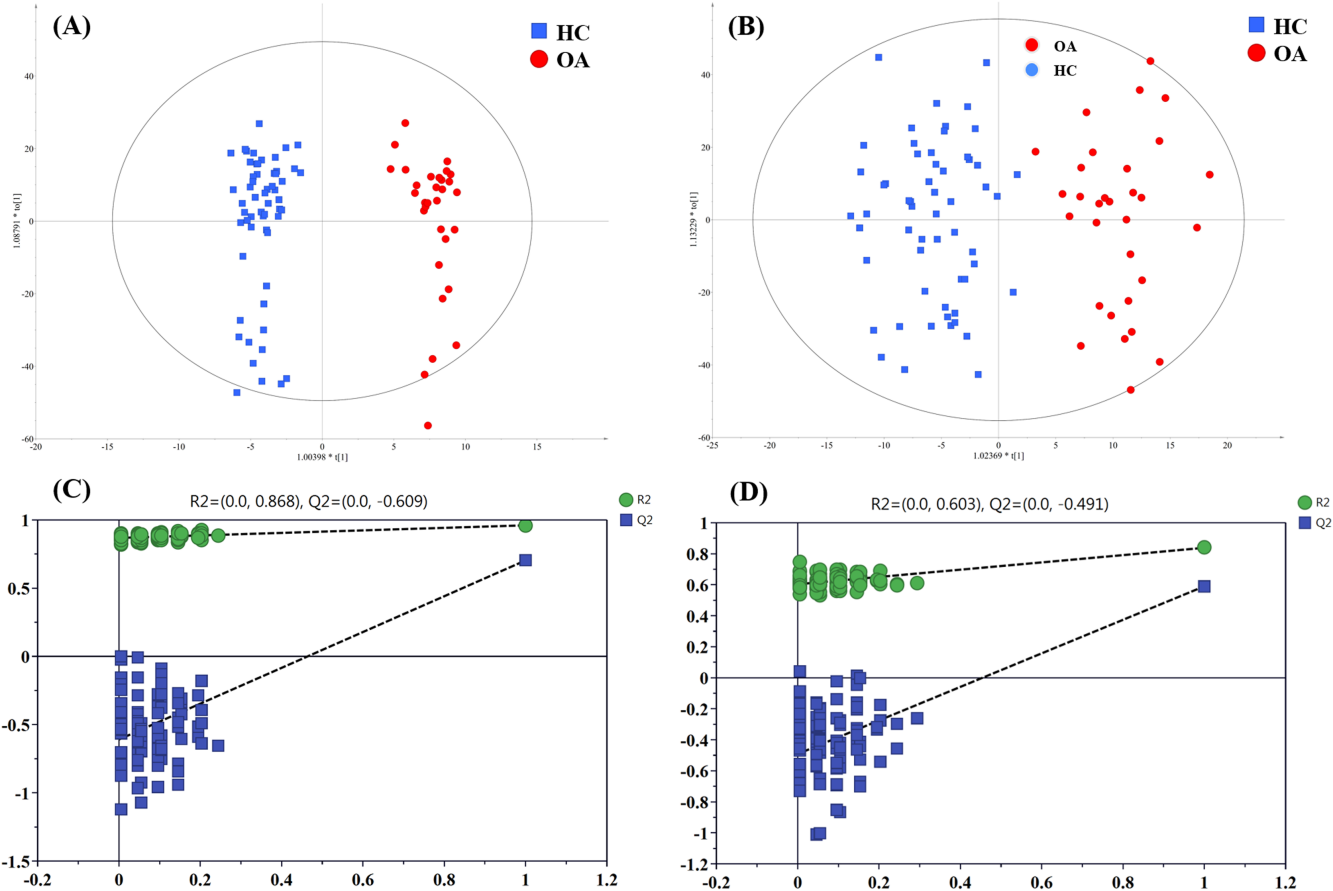
The OPLS-DA 3D score plots based on OA and HC groups samples in (A) positive and (B) negative ionization modes. The corresponding permutation validation plots from (C) positive and (D) negative ionization modes. Red and green color represent OA and HC samples, respectively.

To figure out the metabolites differentiating OA patients from control subjects, the supervised statistical model based on OA and HC groups was conducted. As shown in Fig. 4, a distinct separation between the two groups can be observed with *R*^*2*^*Y* and *Q*^*2*^ values were 0.961 and 0.703 in ESI+ (Fig. 4A), and 0.838 and 0.590 in ESI− (Fig. 4B), suggesting metabolites have been surely changed in OA patients. In the meantime, permutation tests with 100 random iterations were conducted for the purpose of model validation. As presented in Figs. 4C and 4D, their corresponding permutation results were encouraging as all permuted *R*^*2*^ and *Q*^*2*^ values were lower than the original points to the right and that the intercept of *Q*^*2*^ was below zero, implying the feasibility of the constructed model (Yang et al., 2018b).

The influence of the metabolites on the group separation was signified according to VIP value. And 14 significantly changed metabolites was screened out based on VIP value > 4 and significant *p* value < 0.05, including eight kinds of phosphatidylcholine (PC) species, unknown m/z 239.091, *p*-cresol sulfate (*p*-CS), unknown m/z 195.101, 12-hydroperoxy-5,8,10,14-eicosatetraenoic acid (12-HETE), cis-vaccenic acid (CVA) and perfluorooctane sulfonic acid (PFOS), and their detailed information provided in Table 3.

**Table 3:** Discriminative serum metabolites between OA and HC groups, as well as between OA and non-OA groups.

NO	Metabolites	HMDB ID	Mode	RT (min)	Extract mass	OA *vs* HC				OA *vs* non-OA			
						VIP	FC	*p* value	AUC	VIP	FC	*p* value	AUC
1	PC (16:0/22:6)	HMDB0007991	ESI+	8.36	806.571	20.61	1.18	0.015	0.658	26.47	1.43	0.003	0.749
2	Unknown m/z 239.091	HMDB0030254	ESI−	1.87	239.091	19.62	2.19	<0.001	0.774	15.50	1.91	0.014	0.708
3	PC (18:1/18:2)	HMDB0008072	ESI+	9.15	784.586	18.89	1.28	0.008	0.673	18.88	1.48	0.016	0.706
4	PC (16:0/18:1)	HMDB0007972	ESI+	10.33	760.587	16.97	1.13	0.027	0.643	/	/	/	/
5	PC (18:0/22:6)	HMDB0008057	ESI+	10.42	834.602	15.92	1.40	0.002	0.706	14.39	1.68	0.002	0.761
6	PC (18:0/18:0)	HMDB0008036	ESI+	11.93	812.617	15.59	1.52	<0.001	0.715	7.42	1.37	0.041	0.674
7	PC (20:4/16:1)	HMDB0008463	ESI+	7.70	802.537	12.99	1.70	<0.001	0.720	8.79	1.82	0.006	0.689
*8*	*p*-Cresol sulfate	HMDB0011635	ESI−	1.75	187.005	9.28	1.72	0.042	0.632	/	/	/	/
9	Unknown m/z 195.101	HMDB0030984	ESI−	1.87	195.101	8.98	2.12	<0.001	0.768	7.18	1.88	0.015	0.705
10	PC (22:6/18:0)	HMDB0008727	ESI+	11.76	834.598	7.37	1.34	0.040	0.634	/	/	/	/
11	12-hydroperoxy-5,8,10,14-eicosatetraenoic acid	HMDB0062287	ESI−	2.63	319.226	6.59	0.46	<0.001	0.776	/	/	/	/
12	PC (22:6/20:3)	HMDB0008737	ESI+	10.30	856.582	5.28	1.23	0.021	0.650	7.71	1.54	0.008	0.722
13	Cis-vaccenic acid	HMDB0040219	ESI+	4.24	282.279	4.46	0.44	<0.001	0.774	/	/	/	/
14	Perfluorooctane sulfonic acid	HMDB0059586	ESI−	2.31	498.928	4.19	1.51	0.003	0.693	8.32	1.76	0.004	0.742
15	PC (20:4/18:0)	HMDB0008431	ESI+	10.70	810.602	/	/	/	/	20.92	1.27	0.002	0.757
16	Docosahexaenoic acid	HMDB0002183	ESI−	3.48	327.231	/	/	/	/	14.54	1.51	0.039	0.676
17	PC (18:1/22:6)	HMDB0008123	ESI+	10.67	832.583	/	/	/	/	9.15	1.16	0.025	0.689
18	Phenylalanine	HMDB0000159	ESI−	1.10	164.070	/	/	/	/	7.41	0.75	0.012	0.713
19	PC (22:6/18:2)	HMDB0008730	ESI+	7.59	830.569	/	/	/	/	7.03	1.70	0.005	0.733
20	Indoxyl sulfate	HMDB0000682	ESI−	1.65	212.000	/	/	/	/	5.79	1.59	0.010	0.716

**Note:**

The importance in the projection (VIP) was acquired from OPLS-DA models with a threshold of 4.0; The fold change (FC) refers to the ratio of the average concentrations of metabolites in two groups of samples; *p* value was calculated by Kruskal-Wallis test with a threshold of 0.05; The area under the curve (AUC) was calculated using ROC analysis.

#### Metabolites for differential diagnosis

**Figure 5:**
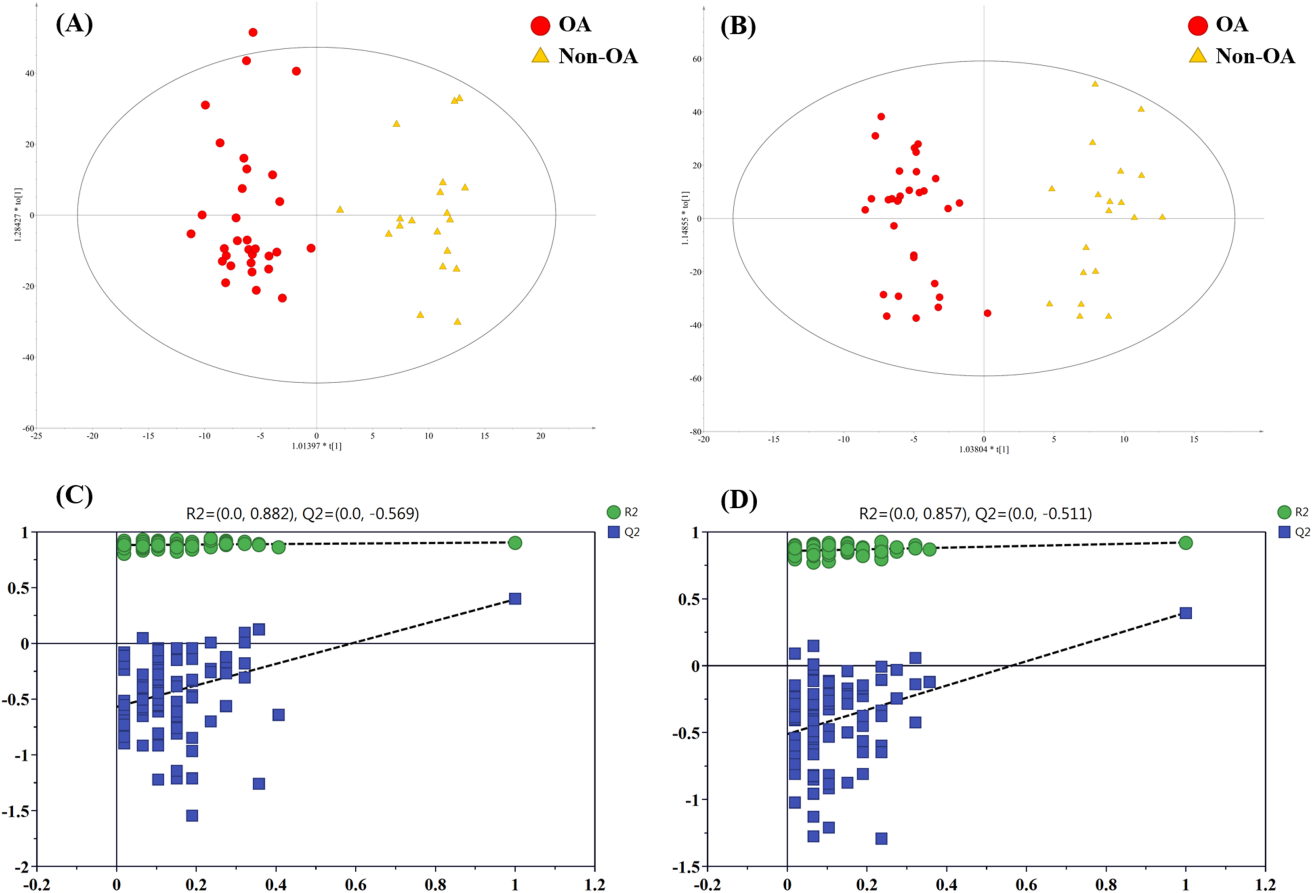
The OPLS-DA 3D score plots based on OA and non-OA samples in (A) positive and (B) negative ionization modes. The corresponding permutation validation plots from (C) positive and (D) negative ion mode models. Red and yellow color represent OA and non-OA samples, respectively.

In order to pick out the metabolites which could be used for differentiating OA patients from non-OA subjects, another pair-wise groups comparison based on OA and non-OA samples was executed. The OPLS-DA 3D score plots were illustrated in Figs. 5A and 5B, still displaying a favorable separation between the two groups. The model parameters values were desirable as follows: *R*^*2*^*Y* = 0.906 and *Q*^*2*^ = 0.396; *R*^*2*^*Y* = 0.921 and *Q*^*2*^ = 0.396, in ESI+ and ESI−, respectively. Thus, it is speculated that there was presence of differential biochemical changes in OA patients when compared with the patients with other types of arthritis. Simultaneously, the corresponding permutation tests results of the models were depicted in Figs. 5C and 5D. All permuted *R*^*2*^ and *Q*^*2*^ values were lower than the original points to the right and that the intercept of *Q*^*2*^ was below zero, indicating the above OPLS-DA models were non-overfitting and valid.

In addition, according to VIP value > 4 and *p* value < 0.05, a total of 15 differentially regulated metabolites were retained and their detailed information were listed in Table 3, including nine members of PC species, unknown m/z 239.091, unknown m/z 195.101, PFOS, docosahexaenoic acid (DHA), phenylalanine (Phe) and indoxyl sulfate.

### Optimization of potential biomarkers

The aforesaid results suggested that the differential biochemical metabolites could be used as unique diagnostic biomarker. ROC curves were established based on the OA-related metabolites to test single metabolite’ diagnostic effectiveness, and their corresponding AUC values were illustrated in Table 3. Every metabolic candidate had an AUC value which exceeded 0.6.

To better understand how multiple metabolites collectively distinguish the OA and HC/non-OA groups, stepwise logistic regression models were built based on above 14/15 candidates, respectively. A group of seven candidates were picked out according to *p* < 0.05 in the logistic regression to diagnose OA from HC groups, namely unknown m/z 239.091 (standardized (std) β = 3.373, *p* = 0.005), PC (18:0/22:6) (std β = 0.132, *p* = 0.019), PC (18:0/18:0) (std β = 0.135, *p* = 0.004), *p*-CS (std β = 0.404, *p* = 0.008), unknown m/z 195.101 (std β = −15.237, *p* = 0.006), 12-HETE (std β = −1.798, *p* = 0.003), CVA (std β = −2.008, *p* = 0.010). And the corresponding logistic regression model was: Logit (P) = −7.444 + 3.373 * log (unknown m/z 239.091) + 0.132 * log (PC (18:0/22:6)) + 0.135 * log (PC (18:0/18:0)) + 0.404 * log (*p*-CS) −15.237 * log (Unknown m/z 195.101) −1.798 * log (12-HETE) −2.008 * log (CVA), with an overall correct percentage of 90.9%. The AUC value of the integrated seven potential biomarkers reached 0.978, with a sensitivity, specificity and Youden index of 96.7%, 93.0%, and 0.898, respectively.

**Figure 6:**
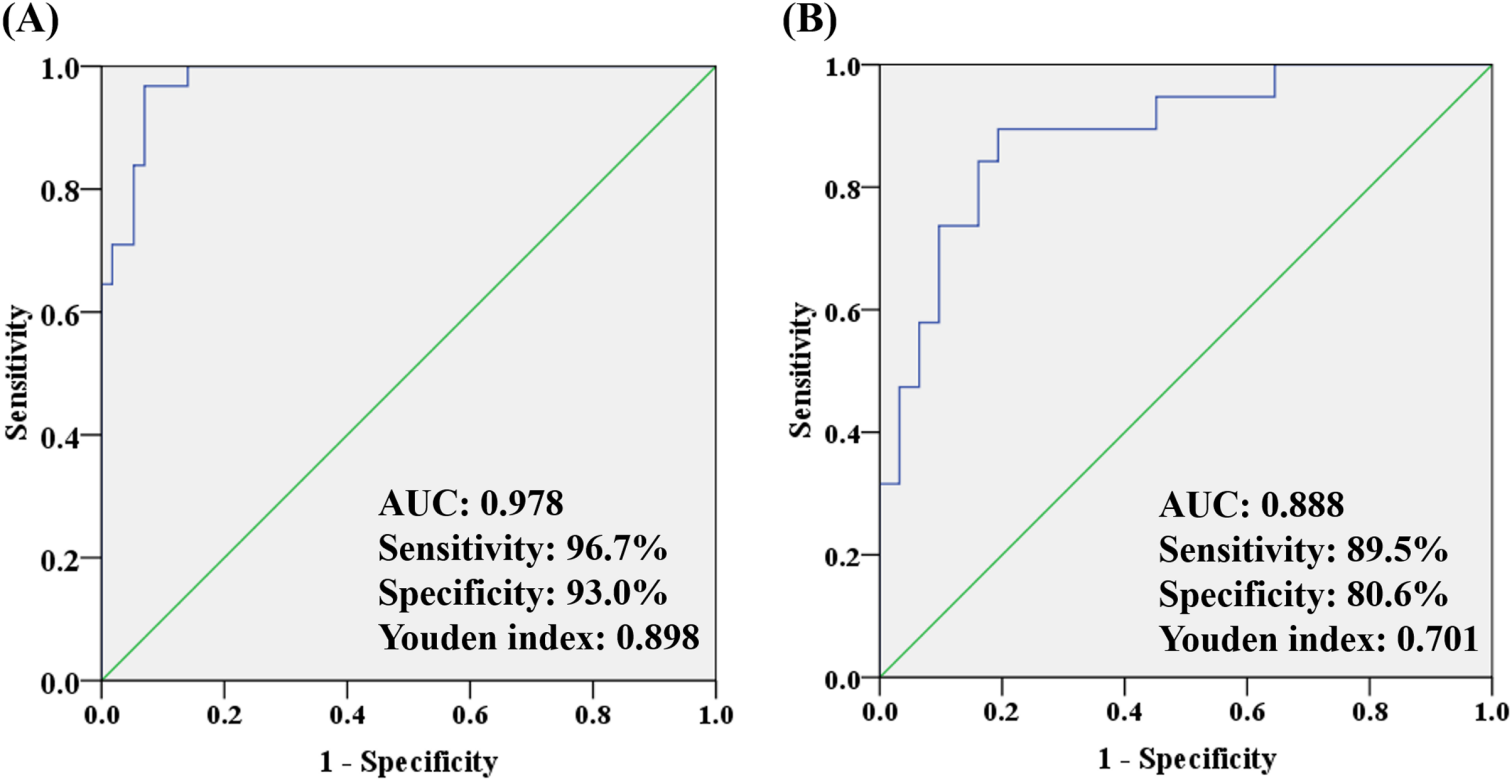
The ROC analysis by combining (A) seven potential biomarkers for discriminating OA and HC groups and (B) three potential biomarkers for discriminating OA and non-OA groups.

In the same way, three candidates were selected with a criterion of *p* < 0.05 in the logistic regression to differentiate OA from non-OA groups, namely unknown m/z 239.091 (standardized (std) β = −0.080, *p* = 0.022), PC (18:0/18:0) (std β = −0.087, *p* = 0.007) and Phe (std β= 0.930, *p* = 0.012). And the corresponding logistic regression model was: Logit (P) = −3.088 − 0.080 * log (unknown m/z 239.091) −0.087 * log (PC(18:0/18:0)) − 0.930 * log (Phe), with an overall correct percentage of 82.0%. Likewise, the integrated three potential biomarkers had a great AUC value of 0.888, with sensitivity of 89.5%, specificity of 80.6% and Youden index of 0.701. And the ROC curves of the combined two panels were plotted in Fig. 6. It was noteworthy that two panels’ AUC values reached 0.978 and 0.888, respectively, yielding greater diagnostic value than most of the single candidate whose AUC value were lower than 0.8 (Table 3).

### OA-related metabolic pathways

**Figure 7:**
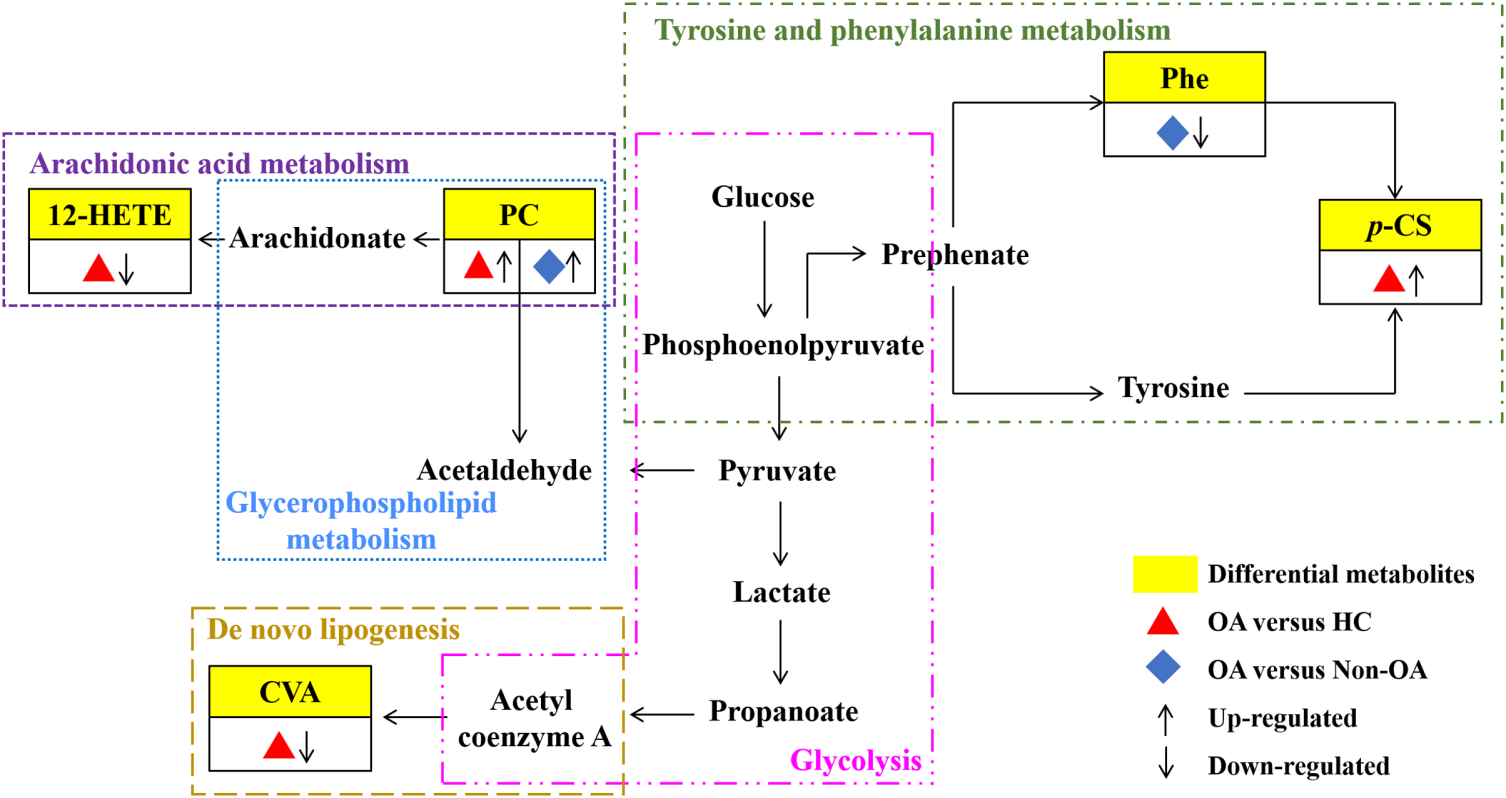
Seven metabolic network of OA potential biomarkers according to pathway analysis. 12-HETE, 12-hydroperoxy-5,8,10,14-eicosatetraenoic acid; PC, phosphatidylcholine; Phe, phenylalanine; *p*-CS, *p*-Cresol sulfate; CVA, cis-vaccenic acid.

Our results have reflected distinctively differential metabolites occurred in OA, indicating that metabolic biological networks may be subjected. And further metabolic pathway analysis is needed to better clarify the holistic status of the altered metabolic markers and explore possible disturbed pathways. The metabolic pathway analysis of the above potential biomarkers with KEGG database exhibited that OA-induced dys-regulated pathways involved in arachidonic acid metabolism; glycerophospholipid metabolism; *de novo* lipogenesis pathway; tyrosine and Phe metabolism and glycolysis (Fig. 7).

## Discussion

OA is a leading cause of disability worldwide and its early and accurate diagnosis is still hampered due to the lack of sensitive and specific biomarkers. Many studies have been demonstrated that metabolic components have been associated with OA genesis and development (Batushansky et al., 2019; de Sousa et al., 2017). And metabolomics could serve as a vital intermediate tool between basic and clinical research whereby providing potential biomarkers (Atanassova, Panchev & Ivanova, 2010; Spratlin, Serkova & Eckhardt, 2009). In view of this, a global serum unbiased metabolomic analysis using an UPLC-QToF/MS platform was performed in the present study. In our previous study, the combination of reverse phase liquid chromatography (RPLC) and hydrophilic interaction liquid chromatography (HILIC) columns can indeed increase the number of metabolites detected in serum metabolomics study (Gao et al., 2015). However, from the view of simplicity and cost, it is not convenient and practical to use both RPLC and HILIC columns for disease screening and diagnosis in a hospital laboratory. Hereby, RPLC-QToF/MS-based metabolomic workflow was employed just like some other researches that employed a single reverse phase column to separate metabolites in serum and urine samples (Wang et al., 2021; Zhang et al., 2022).

In our study, 20 serum metabolites were found to be related to OA. Among them, the significant reduction of serum 12-HETE was found in OA group as in comparison with HC group. And the serum 12-HETE displayed favorable effectiveness to identify OA from HC as its AUC, sensitivity, specificity and Youden index were 0.776, 84.2%, 58.1% and 0.423, respectively. This ROC analysis result suggested that the serum 12-HETE could be a potential diagnostic maker of OA. Moreover, our results also exhibited that OA patients had markedly higher expression level of PC (18:0/22:6) when compared with non-OA subjects. And the serum PC (18:0/22:6) produced an AUC of 0.761, sensitivity of 74.2%, specificity of 73.7% and Youden index of 0.479, indicating that it may be an important differential diagnostic feature of OA.

Except that, to make it better to apply the OA-related metabolites to predict OA for clinical application, we chose and integrated several potential biomarkers for establishing models. Ultimately, the analysis results of ROC curve in combination with logistic regression revealed that unknown m/z 239.091, PC (18:0/22:6), PC (18:0/18:0), *p*-CS, unknown m/z 195.101, 12-HETE and CVA could be selected as candidate biomarkers to diagnose OA from HC groups. This seven-metabolite panel was identified with an AUC of 0.978, sensitivity of 96.7%, specificity of 93.0% and Youden index of 0.898, which displayed better diagnostic capability than 12-HETE. Furthermore, another three-metabolite panel of unknown m/z 239.091, PC (18:0/18:0) and Phe displayed an AUC of 0.888, sensitivity of 89.5%, specificity of 80.6% and Youden index of 0.791 to discriminate OA from non-OA group, which also showed greater differential diagnostic value than PC (18:0/22:6). Summing up, these two panels might be an excellent performing indicator with respect to OA and they could be useful in laboratory medicine.

According to the metabolic pathway analyses, the results displayed that 12-HETE, the long-chain polyunsaturated fatty acids (LC-FUFAs), is originated by arachidonic acid and is formed *via* 12-lipoxygenase-mediated lipoxygenase oxygenation to participate in arachidonic acid metabolism (Charles-Schoeman et al., 2018). PC transforms into arachidonic acid and acetaldehyde, which not only involve in arachidonic acid metabolism, but also in glycerophospholipid metabolism. CVA, a kind of monounsaturated fatty acid (MUFA), was converted by carbohydrates, proteins and acetyl coenzyme A *via* an endogenous pathway called *de novo* lipogenesis (DNL) (Djoussé et al., 2012; Wu et al., 2011). Phe, the essential aromatic amino acid, can produce tyrosine by hydroxylation in Phe metabolism (Dobrian et al., 2011). And *p*-CS is the degradation product of Phe and tyrosine by intestinal epithelial cell sulfotransferase, involving in the tyrosine and Phe metabolism (Peng et al., 2019) (Fig. 7).

Of course, several potential limitations in this study should not be neglected. Firstly, two unknown features with m/z 195.101 and m/z 239.091 were found in this study. These unknown identities hindered the understanding of their biological roles in OA. Secondly, sample capacity in OA and non-OA groups are not large enough to strongly validate these potential biomarkers. Thus, in order to evaluate the predictive ability of the potential biomarkers, blinded tests should be conducted for prediction and validation. At the same time, further clinical tests with a larger sample capacity of subjects and *in vitro* and *in vivo* experiments are required to confirm our aforesaid findings and hypothesis.

In conclusion, this work revealed the feasibility of UPLC-QToF/MS-based serum untargeted metabolomics to capture the metabolic signature and screen the potential biomarkers of OA. It demonstrated that OA-induced metabolic disturbance involved in lipid metabolism, glucose metabolism and amino acid metabolism, which may point towards potential contributing mechanisms in OA pathogenesis and progression. Moreover, it should be noted that the single metabolite has a certain advantage in OA early and accurate diagnosis with AUC greater than 0.7, but the integrated panel yielded a greater diagnostic value than that of each single one. Thus, the above two metabolic panels toward clinical practice for OA diagnosis may be valuable in the future.

## Supplemental Information

10.7717/peerj.14563/supp-1 Supplemental Information 1 Dataset from LC-MS in ESI positive and negative mode.

## Additional Information and Declarations

### Data Availability

The following information was supplied regarding data availability: The raw measurements are available as a Supplemental File.
